# Evidence of nickel and other trace elements and their relationship to clinical findings in acute Mesoamerican Nephropathy: A case-control analysis

**DOI:** 10.1371/journal.pone.0240988

**Published:** 2020-11-10

**Authors:** Rebecca S. B. Fischer, Jason M. Unrine, Chandan Vangala, Wayne T. Sanderson, Sreedhar Mandayam, Kristy O. Murray

**Affiliations:** 1 Section of Tropical Medicine, Department of Pediatrics, Baylor College of Medicine and Texas Children’s Hospital, Houston, TX, United States of America; 2 Department of Epidemiology & Biostatistics, School of Public Health, Texas A&M University, College Station, TX, United States of America; 3 Department of Plant and Soil Sciences, College of Agriculture, Food, and Environment, University of Kentucky, Lexington, KY, United States of America; 4 Section of Nephrology, Department of Medicine, Baylor College of Medicine, Houston, Texas, United States of America; 5 Departments of Epidemiology and Preventive Medicine and Environmental Health, Southeast Center for Agricultural Health and Injury Prevention, College of Public Health, University of Kentucky, Lexington, KY, United States of America; 6 Section of Nephrology, Department of Medicine, MD Anderson Cancer Center, Houston, Texas, United States of America; 7 William T. Shearer Center for Human Immunobiology, Department of Pediatrics, Baylor College of Medicine and Texas Children’s Hospital, Houston, Texas, United States of America; Chinese Academy of Sciences, CHINA

## Abstract

**Background:**

Although there are several hypothesized etiologies of Mesoamerican Nephropathy (MeN), evidence has not yet pointed to the underlying cause. Exposure to various trace elements can cause the clinical features observed in MeN.

**Methods and findings:**

We measured 15 trace elements, including heavy metals, in renal case-patients (n = 18) and healthy controls (n = 36) in a MeN high-risk region of Nicaragua. Toenails clippings from study participants were analyzed using inductively coupled plasma mass spectrometry. A case-control analysis was performed, and concentrations were also analyzed over participant characteristics and clinical parameters. Nickel (Ni) concentrations were significantly higher in toenails from cases (1.554 mg/kg [0.176–42.647]) than controls (0.208 mg/kg [0.055–51.235]; p<0.001). Ni concentrations correlated positively with serum creatinine levels (p = 0.001) and negatively with eGFR (p = 0.001). Greater Ni exposure was also associated with higher leukocyte (p = 0.001) and neutrophil (p = 0.003) counts, fewer lymphocytes (p = 0.003), and lower hemoglobin (p = 0.004) and hematocrit (p = 0.011).

**Conclusions:**

Low-dose, chronic environmental exposure to Ni is a possible health risk in this setting. Ni intoxication and resulting systemic and renal effects could explain the clinical signs observed during early MeN. This study provides compelling evidence for a role of Ni in the acute renal impairment observed in this MeN high-risk population. Additional work to assess exposure levels in a larger and heterogeneous population, identify environmental sources of Ni and exposure pathways, and evaluate the link between Ni and MeN pathogenesis are urgently needed.

## Introduction

An epidemic of chronic kidney disease of unknown etiology (CKDu) emerged in recent decades in Central America. The most recent estimates attribute 50,000 deaths or more to Mesoamerican Nephropathy (MeN), as it is often called, most notably among working-age individuals who are otherwise healthy ([1]. MeN is distinct from other forms of CKD, and traditional risk factors for kidney disease, such as hypertension, diabetes, and advanced age, are not implicated [2–6]. Perhaps the most perplexing characteristic of the epidemic is its geographic distribution, which appears concentrated in ‘hot spots’ along the Pacific coastal lowlands, extending from Mexico to Panama, but sparing areas inland and on the Atlantic coast [7–11].

As part of a large, ongoing investigation into the etiology of MeN, we established active surveillance for impaired renal function in a region of northwestern Nicaragua with excess kidney-related morbidity and mortality [1, 6]. While previously characterized as an insidious chronic disease, we now understand that an early phase often presents as acute kidney injury (AKI) with neutrophilic leukocytosis, leukocyturia, and anemia, often with mild flu-like illness [12, 13]. Early renal pathology reflects acute tubulointerstitial nephritis with inflammatory infiltrate concentrated at the corticomedullary junction [14]. These observations suggest that the acute renal event is triggered by an exogenous, pro-inflammatory agent. While most patients recover renal function following AKI, approximately 10% are diagnosed with CKDu within the first year [13]. It is yet unknown if the AKI epidemic reflects incident kidney disease (e.g. initiation of the MeN disease process) or acute exacerbations of an already underlying, subclinical CKDu. There is an urgent need to uncover the agent of disease and reveal the source of exposure for this vulnerable population.

Heavy metal toxicity has been proposed as a possible etiology of MeN but has not been adequately examined, and the role of other trace elements have not been explored. We conducted this investigation to ascertain if one or more trace elements, including heavy metals, could be implicated in the etiology of MeN. We measured trace element exposure in a population of agriculture workers with documented high rates of MeN [13, 15, 16]. We employed a case-control analysis to compare exposure levels in individuals with documented acute renal impairment to healthy controls from the same population. Our overall goal is to provide evidence to guide etiologic discovery and other investigations into the devastating, decades-long epidemic of CKDu in Central America.

## Methods

### Data and specimen collection

This study was conducted at a commercial agriculture estate in northwestern Nicaragua, whose primary crop is sugarcane, as part of a large and interdisciplinary investigation into the underlying cause(s) of MeN [12, 15]. The estate is in an area hyperendemic for MeN and is a hypothesized epicenter of the epidemic. The occupational health program monitors workers’ renal function by measuring serum creatinine levels at annual health screenings and through targeted screening of field workers. In addition, all patients are screened during clinical encounters at the estate’s private hospital, which serves as the main source of primary healthcare for workers and their families.

We began hospital-based surveillance for elevated serum creatinine, AKI, and CKD at this site in 2015. Clinicians report all cases of acute (elevated serum creatinine [>1.3 mg/dl for males; >1.1 mg/dl for females] or reduced creatinine clearance [17] [<90 ml/min]) and chronic (CKD diagnoses) renal impairment (Table 1). Acute events are suspected as attributable to MeN by local clinicians if they lack evidence or suspicion of an underlying cause of kidney disease, such as diabetes, hypertension, or congenital cause. This classification is consistent with the current understanding of MeN and Pan American Health Organization guidance [2, 4, 12, 13]. Suspected case-patients receive overnight hydration and electrolytes; almost all fail renal recovery during that short time and are invited to enroll in our MeN cohort. The study population is well described; patients are classified as having AKI if they meet established AKIN criteria [12–14, 18]. Cohort participants with acute renal events during May 2016-Sep 2017 are the source of cases for this analysis.

**Table 1:** Definitions.

Normal/Healthy	Normal serum creatinine (≤1.3 mg/dL for males or ≤1.1 mg/dL for females)
Incident AKI	Presentation with serum creatinine *increased* (≥0.3 mg/dL or ≥1.5-fold) over baseline
	• Baseline referenced to most recent normal measurement within 1 year prior to AKI event
Renal Recovery	Return to normal serum creatinine (>1.3 mg/dL for males or >1.1 mg/dL for females)
	• Maintained for ≥1 year following AKI event
Incident CKD	Diagnosis by local nephrologist following ≥3 months of
	• reduced glomerular filtration rate (<60mL/min/1.73m2), calculated from serum creatinine, or
	• functional or structural renal alteration, ascertained by blood, urine, or imaging studies
MeN Diagnosis	Diagnosis by local nephrologist with renal disease (acute or chronic) that cannot reasonably be attributed to any known cause or underlying comorbidity, such as hypertension, diabetes, old age, obesity, or congenital deformity

A convenience sample of agricultural workers from the same estate were enrolled as healthy counterparts during occupational health screenings in Oct 2016, comprising our “control cohort”. Controls in this analysis had normal serum creatinine and lacked history of renal disease, assessed by personal interview and medical record review. We achieved a 1:2 ratio of cases to controls. This research was reviewed and approved by the Comité Instucional de Revisión Ética (CIRE) del Minsterio de Salud (MINSA) de Nicaragua, the Medical Director of Hospital Alfredo Pellas Chamorro, and the Baylor College of Medicine Institutional Review Board (H-36498). Written, informed consent was provided by all study participants.

Clinical features, demographics, and laboratory data (blood chemistry, hematology, and urine analysis) from the hospital encounter during the acute renal event (for cases) or health screening visit (for controls) was recorded by clinical staff. To characterize change from baseline, most recent serum creatinine and hematocrit levels and leukocyte count measured prior to enrollment were abstracted from medical records. Participants were contacted 3 months following enrollment for toenail samples, so that a collected nail fragment reflected exposures temporally associated with their acute renal event and enrollment visit. Clippings from the great toe, using clean, stainless steel nail clippers (new per each participant, purchased as a lot [Fromm International, United States]) were deposited directly into paper specimen envelopes and transported to the University of Kentucky for analysis.

### Measurement of elements in toenail specimens

Nails were prepared and analyzed as follows [19]. They were cleaned by sonication in 1 mL of trace metal grade acetone for 15-min, followed by sonication in 1mL 1% Citranox metal-free detergent for 15-min, and two repetitions of sonication in 1mL 18.2 MΩ deionized (DI) water for 15-min each. Nails were dried at 60°C to a constant weight, microwave digested in ultra-pure HNO_3_ and H_2_O_2_, diluted with DI water, and analyzed by inductively coupled plasma mass spectrometry for 15 elements: nickel (Ni), aluminum (Al), vanadium (V), manganese (Mn), iron (Fe), cobalt (Co), copper (Cu), zinc (Zn), arsenic (As), selenium (Se), cadmium (Cd), mercury (Hg), lead (Pb), uranium (U), and chromium (Cr). To account for variability in nail masses, the sample-specific method detection limit (MDL) was calculated for each specimen as: three times the standard deviation of the digestion blanks times the dilution factor divided by the nail dry mass. Any value less than the MDL reflects a measurement indistinguishable from background noise for the element in the specimen, a common problem with small specimen size in toxicology limiting statistical analysis; thus, in accordance with standard practice, measurements <MDL were excluded from analysis. Reference limits for toenail element concentrations are provided in S1 Table. Quality control procedures followed U.S. EPA method 6020a [20].

### Statistical analysis

We conducted a case-control analysis of trace element exposures as potential contributors of acute renal impairment in the setting of MeN. Trace element composition in toenail clippings from 3 months subsequent to enrollment were considered evidence of exposure temporally proximal to enrollment. Each trace element concentration (if ≥MDL) is reported in terms of mg of metal/kg of dry toenail mass. Frequencies (n [%]) are reported for categorical variables, and continuous variables are reported as median (range). We compared toenail concentrations from case-patients to those from controls by quantile and Poisson regressions. We report p-values for comparisons of categorical variables using Pearson’s chi-square or Fisher’s exact test, and quantile regression for difference in medians for continuous variables. Correlations are reported using Spearman’s correlation coefficient between continuous elements and using Goodman and Kruskal’s gamma between ordinal elements.

Using linear regression on log_10_-transformed data, we assessed association between element concentrations and serum creatinine. For any element correlated with serum creatinine levels or associated with case status, we assessed association with clinical laboratory parameters (e.g. hematocrit, leukocyte count).

Finally, we created two exposure groupings: (1) toenail concentration ≥ the median, vs < the median, and (2) concentrations ≥75^th^-percentile (i.e. the highest exposure quartile), vs <25^th^-percentile (i.e. the lowest exposure quartile). To estimate risk of exposure by select demographic characteristics, we report risk ratios with 95% confidence intervals (RR [95%CI]). Next, we estimate risk of AKI and of MeN case status following Ni exposure and report RRs over the same two exposure groupings.

Data management and analyses were performed in Stata 15 (StataCorp, College Station, USA); statistical tests are two-sided and considered significant at p<0.05.

## Results

Toenail clippings were collected from 18 individuals with acutely elevated serum creatinine (median age 26.9 years [range 20.0–40.0]) and no prior diagnosis of CKD, comprising the renal cases for this analysis (Table 2). Control participants (n = 36) were similarly aged (29.5 years [19.0–59.0]; p = 0.229) with normal creatinine. Participants had worked at the agriculture estate a median of 5 years, similar for cases (4.0 [0.03–18.0]) as controls (6.6 [0.8–35.9]; p = 0.226). Although highly variable, toenail masses did not differ by case (4.09g [0.16–30.36]) or control (2.76g [0.55–12.35]; p = 0.235) status.

**Table 2:** Characteristics of renal case patients and controls (N = 54).

				ALL	Case	Control	p-value
			N*	N (%)	18 (33.3%)	36 (66.7%)	
Sex							
	Male		54	49 (90.7%)	14 (77.8%)	35 (97.2%)	0.038
	Female			5 (9.3%)	4 (22.2%)	1 (2.8%)	
Age (years)							
	<25		54	17 (31.5%)	7 (38.9%)	10 (27.8%)	0.733
	25–29			13 (24.1%)	5 (27.8%)	8 (22.2%)	
	30–34			15 (27.8%)	4 (22.2%)	11 (30.6%)	
	≥35			9 (16.7%)	2 (11.1%)	7 (19.4%)	
Number of Years Working at Current Employer							
	= <2		51	10 (19.6%)	5 (33.3%)	5 (13.9%)	0.184
	3 to 6			18 (35.3%)	6 (40%)	12 (33.3%)	
	7 to 10			11 (21.6%)	3 (20%)	8 (22.2%)	
	>10			12 (23.5%)	1 (6.7%)	11 (30.6%)	
**Clinical Status at Enrollment**							
	AKI Stage						
		AKIN Criteria not Met	48	32 (66.7%)	2 (11.1%)	30 (100%)	<0.001
		Stage 1		5 (10.4%)	5 (27.8%)	0 (0%)	
		Stage 2		7 (14.6%)	7 (38.9%)	0 (0%)	
		Stage 3		4 (8.3%)	4 (22.2%)	0 (0%)	
**Clinical Status at Baseline^**							
	Elevated Creatinine (>1.3 mg/dL male, >1.1 mg/dL female)		48	0 (0%)	0 (0%)	0 (0%)	n/a
	Hematocrit Anemia (<38.8% male, <34.9% female)		31	1 (3.2%)	0 (0%)	1 (5.9%)	1.000
				Mean SD (Range) or Median (Range)			
Age (years)			54	29 (19, 59)	26.9 (20, 40)	29.5 (19, 59)	0.229
Number of Years Working Current Employer			54	4.7 (0.03, 35.9)	3.6 (0.03, 18.0)	6.6 (0.8, 35.9)	0.226
**Changes from Baseline^**							
	Baseline Serum Creatinine (mg/dL)		48	1.0 (0.7, 1.3)	1.0 (0.7, 1.3)	1.0 (0.8, 1.2)	1.000
		Change (mg/dL)	48	0.1 (-0.2, 3.3)	1.2 (0.2, 3.3)	0 (-0.2, 0.5)	<0.001
		Percent Change (%)	48	10.0 (-22.2, 330.0)	139.2 (16.7, 330.0)	0 (-22.2, 62.5)	<0.001
		Ratio (Enrollment:Baseline)	48	1.1 (0.8, 4.3)	2.4 (1.2, 4.3)	1.0 (0.8, 1.6)	<0.001
	Baseline Hematocrit (%)		31	45 (38, 49)	45.5 (39, 49)	44 (38, 48)	0.594
		Change (%)	30	-0.6 (-12, 8)	-9.0 (-12, -3.6)	1.0 (-1, 8)	<0.001
		Percent Change (%)	30	-1.4 (-25.5, 19.5)	-20.5 (-25.5, -9.2)	2.1 (-2.3, 19.5)	<0.001
		Ratio (Enrollment:Baseline)	30	0.99 (0.75, 1.2)	0.79 (0.75, 0.91)	1.02 (0.98, 1.2)	<0.001
	Days Elapsed from Baseline		48	180 (29, 361)	92.5 (29, 188)	255.5 (57, 361)	<0.001
	Baseline eGFR		48	101.3 (69.0, 127.8)	102.5 (69.0, 127.8)	101.3 (75.7, 123.4)	0.900
	Baseline Creatinine Clearance (mL/min)		48	106.5 (70.8, 189.0)	110.6 (70.8, 189.0)	103.7 (88.6, 172.5)	0.280
		Decrease from Baseline (mL/min)	48	9.6 (-32.5, 113.4)	57.9 (10.1, 113.4)	0 (-32.5, 38.2)	<0.001

*Number of participants on whom data was available.

^Most recent laboratory data recorded within the prior year.

There was no appreciable renal dysfunction in cases or controls at baseline (Table 2), determined retrospectively using available clinic records from annual screenings prior to the harvest. Serum creatinine measurements available on 48 subjects (18 cases; 30 controls) were normal at 6 months prior (180 days [29–361]). Baseline hematocrits on 31 subjects were also normal, save one control male with borderline (Hct 38%) anemia. At acute presentation, creatinine values in case-patients reflected a 2.4-fold increase over baseline, and hematocrit levels had dropped substantially from 45.5% (range 39%-49%) at baseline to 36.0% (range 28%-41%). Almost all (89%; n = 16) cases were characterized as having AKI by AKIN [18] criteria and did not recover normal renal function with overnight IV-saline. No changes from baseline were observed in controls.

### Trace elements in toenails

**Fig 1:**
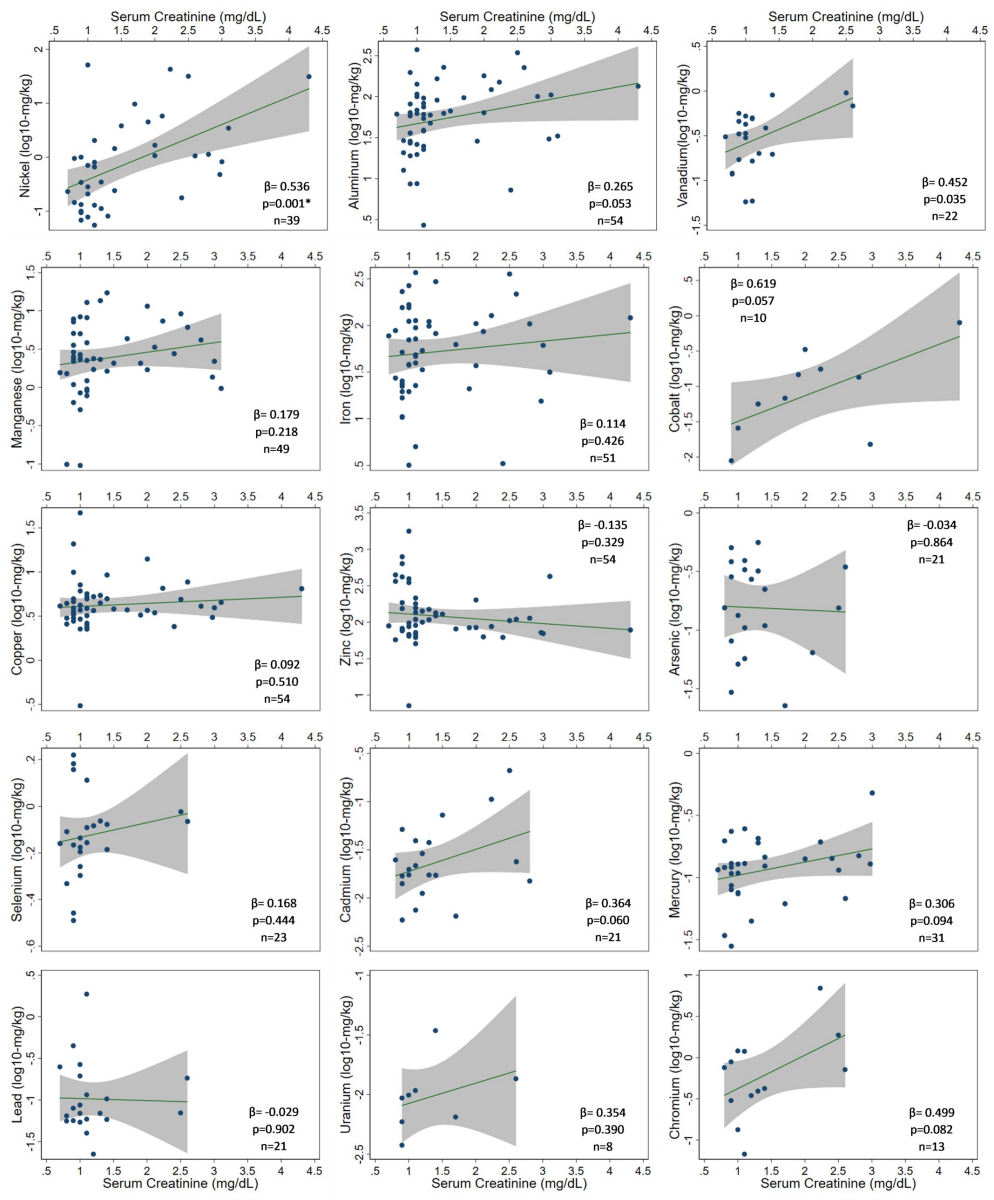
Relationship between toenail trace element concentrations (log_10_-mg/kg dry nail mass) and serum creatinine level (mg/dL).

Compared to control participants, toenails from cases had higher concentrations of Ni (1.55 mg/kg [0.18–42.65] vs 0.21 mg/kg [0.06–51.24]; p<0.001), Al (98.94 mg/kg [7.25–344.34] vs 50.92 mg/kg [2.71–374.73]; p = 0.020) and V (0.79 mg/kg [0.20–0.95] vs 0.32 mg/kg [0.06–0.56]; p = 0.050). In all, 6 elements had sufficient measurements for further analysis (≥MDL): Al (100%), Cu (100%), Zn (100%), Ni (72.2%), Mn (90.7%), and Fe (94.4%) (S2 Table). Of these, a dose-response relationship with serum creatinine was observed only with Ni (p = 0.001) (Fig 1). A similar pattern emerged for V (p = 0.035); however, a limited number of measurements ≥MDL (n = 22), resulting in unknown concentrations for subjects with the highest creatinine levels, limits any interpretation of its role in this disease (Table 3). With respect to Ni, cases predominated the higher exposure group:77.8% (n = 14) had Ni above the study median (0.703 mg/kg), compared to only 23.8% (n = 5) of controls (p = 0.001) (Table 4, S2 and S3 Tables). The higher exposure conveyed a near-4-fold risk of renal case status (RR 3.7 [1.5–9.3]).

**Table 3:** Toenail trace element concentrations (mg/kg dry nail mass), by quartile, for renal case patients and controls.

			Control	Case	ALL			No AKI	AKI	ALL	
** **		N	36 (66.7%)	18 (33.3%)	54 (100%)	p-value	N	32 (66.7%)	16 (33.3%)	48 (100%)	p-value
**Ni**	Range	39	0.05–51.24	0.18–42.65	0.06–51.24		35	0.06–51.24	0.18–42.65	0.06–51.24	
** **	25^th^ Percentile		0.10	0.82	0.15	0.002*		0.09	1.06	0.13	0.001*
** **	50^th^ Percentile		0.21	1.55	0.70	<0.001*		0.23	2.56	0.82	0.015*
** **	75^th^ Percentile		0.70	5.78	2.04	0.471		0.70	7.72	3.45	0.586
**Al**	Range	54	2.71–374.73	7.25–344.34	2.71–374.73		48	8.62–374.74	7.25–344.34	7.254–374.73	
** **	25^th^ Percentile		25.54	59.64	28.67	0.005*		27.90	48.43	29.81	0.019*
** **	50^th^ Percentile		50.92	98.94	61.81	0.020*		56.33	102.97	61.81	0.064
** **	75^th^ Percentile		82.23	150.57	100.67	0.082		78.99	165.26	106.39	0.051
**V**	Range	22	0.06–0.56	0.20–0.95	0.06–0.95		17	0.06–0.56	0.68–0.95	0.06–0.95	
** **	25^th^ Percentile		0.17	0.44	0.17	0.002*		0.17	0.68	0.20	0.002*
** **	50^th^ Percentile		0.32	0.79	0.33	0.050*		0.31	0.90	0.34	0.002*
** **	75^th^ Percentile		0.46	0.93	0.50	<0.001*		0.43	0.95	0.52	<0.001*
**Mn**	Range	49	0.10–13.60	0.97–17.19	0.10–17.19		46	0.10–13.60	0.97–17.19	0.10–17.19	
** **	25^th^ Percentile		1.02	2.07	1.51	0.062		0.95	2.07	1.36	0.072
** **	50^th^ Percentile		2.31	2.76	2.32	0.735		2.31	3.35	2.32	0.310
** **	75^th^ Percentile		4.43	6.10	5.03	0.344		3.56	7.36	4.33	0.214
**Fe**	Range	51	3.18–368.25	3.31–355.71	3.18–368.25		45	3.18–266.91	3.31–355.71	3.18–355.71	
** **	25^th^ Percentile		22.69	33.51	23.63	0.390		22.69	31.60	23.63	0.607
** **	50^th^ Percentile		49.98	81.89	61.22	0.226		49.98	86.16	61.22	0.202
** **	75^th^ Percentile		110.05	120.73	110.81	0.865		98.33	127.50	110.05	0.692
**Co**	Range	10	0.01–0.06	0.02–0.80	0.01–0.80		10	0.01–0.06	0.015–0.80	0.01–0.80	
** **	25^th^ Percentile		0.01	0.07	0.03	0.313		0.01	0.07	0.03	0.313
** **	50^th^ Percentile		0.03	0.15	0.10	0.590		0.03	0.15	0.10	0.590
** **	75^th^ Percentile		0.06	0.33	0.18	0.288		0.06	0.33	0.18	0.269
**Cu**	Range	54	0.31–46.91	2.41–14.06	0.31–46.91		48	0.31–46.91	2.41–14.06	0.31–46.91	
** **	25^th^ Percentile		2.85	3.67	3.06	0.083		3.11	3.56	3.28	0.261
** **	50^th^ Percentile		3.79	4.31	3.91	0.341		4.15	4.01	4.11	0.914
** **	75^th^ Percentile		4.97	6.47	5.21	0.425		5.09	6.51	5.23	0.307
**Zn**	Range	54	7.17–1773.46	62.08–425.09	7.17–1773.46		48	7.17–1773.46	62.08–425.09	7.167–1773.46	
** **	25^th^ Percentile		74.28	78.26	76.39	0.852		74.28	75.18	74.28	0.854
** **	50^th^ Percentile		98.79	96.36	98.79	0.869		104.25	85.99	98.79	0.410
** **	75^th^ Percentile		200.06	129.06	156.69	0.527		167.89	118.23	151.98	0.414
**As**	Range	21	0.03–0.56	0.02–0.35	0.02–0.56		19	0.03–0.56	0.02–0.35	0.02–0.56	
** **	25^th^ Percentile		0.08	0.06	0.08	0.847		0.08	0.06	0.06	0.801
** **	50^th^ Percentile		0.27	0.13	0.16	0.317		0.14	0.16	0.16	0.997
** **	75^th^ Percentile		0.38	0.22	0.33	0.167		0.32	0.22	0.32	0.411
**Se**	Range	23	0.32–1.66	0.65–0.95	0.32–1.66		19	0.32–1.66	0.65–0.95	0.32–1.66	
** **	25^th^ Percentile		0.55	0.74	0.64	0.112		0.60	0.65	0.64	0.925
** **	50^th^ Percentile		0.70	0.85	0.73	0.631		0.72	0.86	0.73	0.402
** **	75^th^ Percentile		0.87	0.90	0.87	0.993		0.83	0.95	0.86	0.597
**Cd**	Range	21	0.01–0.05	0.01–0.21	0.006–0.21		19	0.006–0.04	0.006–0.21	0.006–0.21	
** **	25^th^ Percentile		0.01	0.02	0.015	0.576		0.01	0.02	0.01	0.968
** **	50^th^ Percentile		0.02	0.03	0.02	0.513		0.02	0.02	0.02	0.828
** **	75^th^ Percentile		0.03	0.09	0.04	0.201		0.02	0.11	0.03	0.122
**Hg**	Range	31	0.03–0.25	0.06–0.48	0.03–0.48		28	0.03–0.25	0.06–0.48	0.03–0.48	
** **	25^th^ Percentile		0.08	0.12	0.08	0.284		0.08	0.12	0.08	0.292
** **	50^th^ Percentile		0.12	0.14	0.12	0.464		0.12	0.14	0.26	0.622
** **	75^th^ Percentile		0.16	0.15	0.15	0.794		0.19	0.15	0.17	0.604
**Pb**	Range	21	0.02–1.88	0.06–0.18	0.02–1.88		16	0.02–0.25	0.07–0.18	0.02–0.25	
** **	25^th^ Percentile		0.06	0.06	0.06	0.506		0.06	0.07	0.06	0.648
** **	50^th^ Percentile		0.07	0.09	0.07	0.666		0.07	0.10	0.07	0.587
** **	75^th^ Percentile		0.19	0.14	0.18	0.902		0.09	0.18	0.11	0.145
**U**	Range	8	0.004–0.01	0.006–0.03	0.004–0.03		7	0.004–0.01	0.006–0.03	0.004–0.03	
** **	25^th^ Percentile		0.006	0.006	0.006	0.912		0.005	0.006	0.006	0.953
** **	50^th^ Percentile		0.009	0.01	0.01	0.479		0.008	0.01	0.01	0.517
** **	75^th^ Percentile		0.01	0.03	0.01	0.062		0.01	0.03	0.01	0.101
**Cr**	Range	13	0.07–1.21	0.34–6.96	0.07–6.96		11	0.07–1.21	0.72–6.96	0.07–6.96	
** **	25^th^ Percentile		0.22	0.42	0.35	0.954		0.32	0.72	0.35	0.532
** **	50^th^ Percentile		0.57	0.72	0.72	0.971		0.41	1.88	0.72	0.172
** **	75^th^ Percentile		1.04	1.88	1.19	0.704		0.97	6.96	1.21	0.053

*Statistically significant at p<0.05.

**Table 4:** Toenail trace element concentrations (mg/kg dry nail mass), proportions by quartile, for renal case patients and controls.

			Control	Case	ALL		RR (95% CI)		No AKI	AKI	ALL		RR (95% CI)
		N	36 (66.7%)	18 (33.3%)	54 (100%)	p-value		N	32 (66.7%)	16 (33.3%)	48 (100%)	p-value	
**Ni**													
** **	>25^th^ Percentile	39	12 (57.1%)	18 (100%)	30 (76.9%)	0.002*	11.9 (5.1, 28.2) ^±^	35	10 (52.6%)	16 (100%)	26 (74.3%)	0.001*	12.2 (5.2, 28.9)^±^
** **	>50^th^ Percentile	39	5 (23.8%)	14 (77.8%)	19 (48.7%)	0.001*	3.7 (1.5, 9.3)	35	4 (21.1%)	14 (87.5%)	18 (51.4%)	<0.001*	6.6 (1.5, 29.1)
** **	>75^th^ Percentile	39	1 (4.8%)	8 (44.4%)	9 (23.1%)	0.006*	2.7 (1.5, 4.7)	35	1 (5.3%)	8 (50%)	9 (25.7%)	0.005*	2.9 (1.5, 5.4)
**Al**													
** **	>25^th^ Percentile	54	24 (66.7%)	16 (88.9%)	40 (74.1%)	0.106	2.8 (0.7, 10.8)	48	23 (71.9%)	14 (87.5%)	37 (77.1%)	0.293	2.1 (0.6, 7.9)
** **	>50^th^ Percentile	54	14 (38.9%)	13 (72.2%)	27 (50%)	0.042*	2.6 (1.1, 6.3)	48	12 (37.5%)	12 (75%)	24 (50%)	0.030*	3.0 (1.1, 8.1)
** **	>75^th^ Percentile	54	5 (13.9%)	9 (50%)	14 (25.9%)	0.008*	2.9 (1.4, 5.8)	48	5 (15.6%)	9 (56.3%)	14 (29.2%)	0.006*	3.1 (1.4, 6.8)
**V**													
** **	>25^th^ Percentile	22	13 (72.2%)	4 (100%)	17 (77.3%)	0.535	3.0 (1.3, 7.1)^±^	17	11 (78.6%)	3 (100%)	14 (82.4%)	1.000	1.9 (0.8, 4.5)^±^
** **	>50^th^ Percentile	22	8 (44.4%)	3 (75%)	11 (50%)	0.586	3.0 (0.4, 25.8)	17	6 (42.9%)	3 (100%)	9 (52.9%)	0.206	6.3 (0.4, 15.4)^±^
** **	>75^th^ Percentile	22	3 (16.7%)	3 (75%)	6 (27.3%)	0.046*	3.0 (1.3, 7.2)	17	2 (14.3%)	3 (100%)	5 (29.4%)	0.015*	15.2 (6.3, 36.8)^±^

*Statistically significant at p<0.05.

^±^Because complete separation was observed, a correction (+ 0.5 to each cell frequency) was utilized to obtain RR estimate.

### Nickel and select clinical parameters

**Fig 2:**
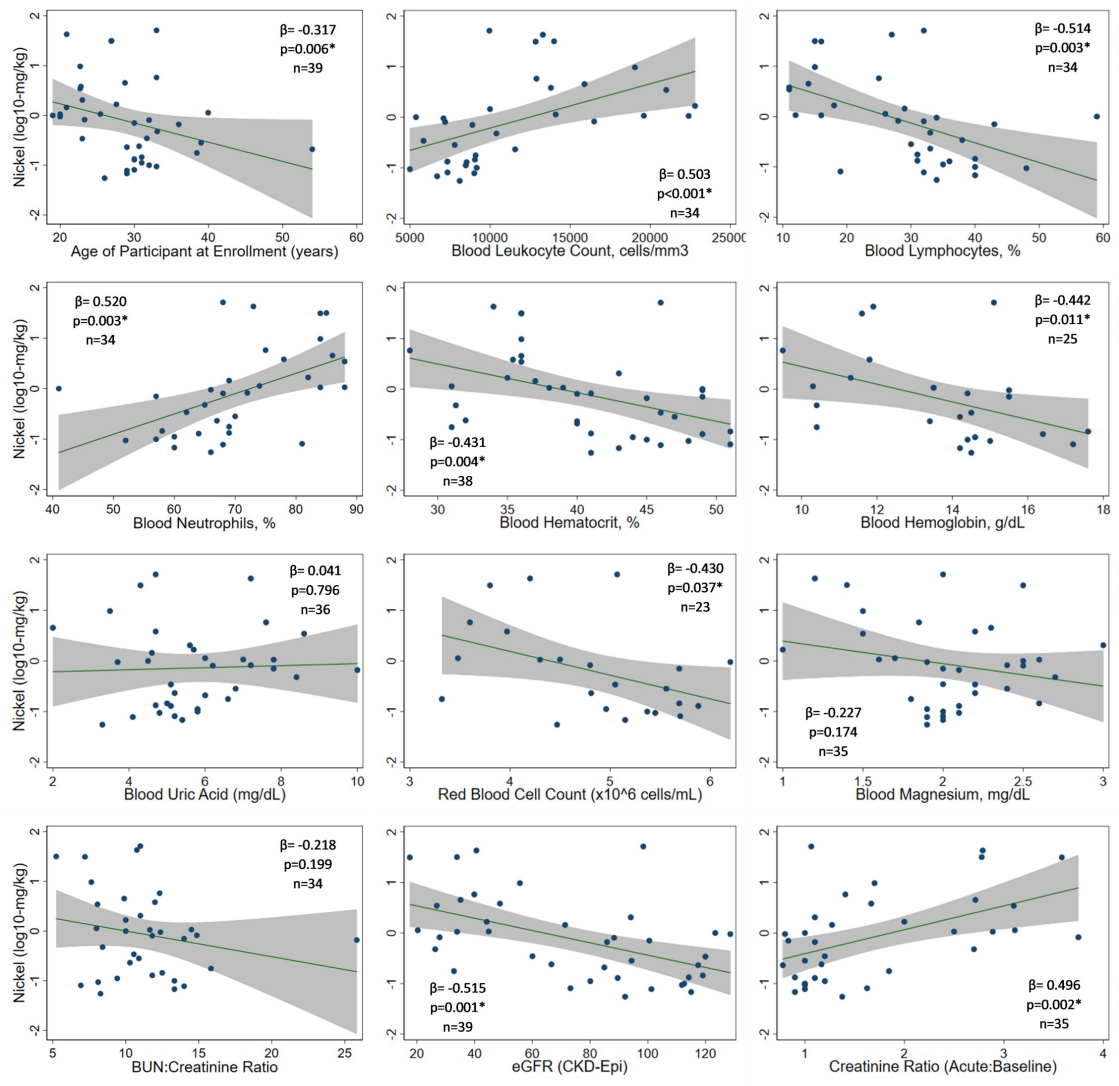
Relationship between toenail nickel concentrations (log10-mg/kg dry nail mass) and select physiologic parameters.

Because of its association with decreased renal function, we compared Ni concentrations to physiologic observations (Fig 2 and S1 Fig). Ni levels correlated with blood leukocyte (p = 0.001) and neutrophil counts (p = 0.003) but were inversely related to lymphocyte counts (p = 0.003), hemoglobin (p = 0.004), hematocrit (p = 0.011), red blood cell counts (p = 0.037), and age (p = 0.006) (Fig 2). Higher Ni concentrations corresponded to lower eGFRs (p = 0.001) and greater fold-increase in serum creatinine over baseline (p = 0.002). Similar analyses of Al and V are presented in S1 and S2 Figs, respectively, but sample measurements limit their interpretation.

We defined an intense Ni-exposure group as the 9 (23.1%) subjects with Ni levels >2.04 mg/kg (75^th^ percentile in this study). Compared to the lowest exposure group (<0.15 mg/kg)., who were overwhelmingly asymptomatic with normal laboratory parameters, the most distinguishing features of intense exposure were elevated creatinine (88.9% vs 0%; p<0.001), moderate leukocytosis (88.9% >12,000 cells/mm^3^ vs 0%; p<0.001), neutrophilia (100% vs 33.3%; p<0.001), anemia (80.0% vs 0%; p = 0.010), low red cell counts (80% vs 0%; p = 0.010), and leukocyturia (77.8% vs 11.1%; p = 0.015) (S3 Table). They also reported back pain (75.0%), abdominal pain (71.4%), fever (66.7%), and nausea +/- vomiting (66.7%). Subjects in the higher exposure groups also tended to be younger: age <30 years corresponded to excess risk of exposures putting them above the Ni median, even after adjusting for case status (RR 2.8 [1.1–6.9]; p = 0.029). No significant differences were observed with respect to sex or number of years working at the agricultural study site. These intensely exposed individuals had a heightened risk of renal event (RR 2.7 [1.5–4.7], compared to lower exposure groups) though risk, even though less precise, is suggested even at lower levels of exposure (RR 11.9 [5.1–28.2] for individuals ≥25%-tile vs those in the lowest quartile).

Although the median toenail Ni concentration in this study population was similar to that for the healthy, rural U.S. reference population (0.79 ug/g; S1 Table), our 75^th^ percentile approximated the 90^th^ percentile from the reference population, suggesting at least some of our study participants face greater Ni exposures [19].

### Nickel exposure and MeN CKD

Two case-patients, and none of the controls, were subsequently diagnosed with CKD attributed to MeN: (1) a 33-yr old male, diagnosed with stage 3 CKD only 3 months after acute presentation, whose toenail Ni concentration was 0.48 mg/kg; and (2) a 32-year old male, diagnosed with stage 3 CKD after 16 months, whose toenail Ni concentration was 0.24 mg/kg (Table 5).

**Table 5:** Characteristics of subjects who developed CKD of unknown etiology.

	Case 1	Case 2
Sex	Male	Male
Age (years)	30.6	33.1
Month of Acute Presentation	June 2016	April 2017
Occupation	Seasonal sugarcane seed cutter; not working in the 1 month prior	Seasonal sugarcane seed cutter; working in the 1 month prior
Healthy Baseline		
Creatinine (mg/dL)	1.2	1.3
Hematocrit (%)	Unknown*	42
Hemoglobin (g/dL)	Unknown*	Unknown*
Leukocytes (cells/mm^3^)	Unknown*	Unknown*
Acute Presentation		
Creatinine (mg/dL)	1.4	2.97
Hematocrit (%)	32.0	31.3
Hemoglobin (g/dL)	Unknown*	10.4
Leukocytes (cells/mm^3^)	Unknown*	10400
AKI Stage	AKIN criteria not met	2
Number of Days from Baseline to		
Acute Visit	30	97
Number of Days from Acute to		
CKD Diagnosis	508	93
CKD Stage	3	3
Concentration of Trace Element in Toenail (mg/kg)		
Nickel	0.24	0.48
Aluminum	62.64	30.47
Vanadium	0.20	<MDL
Manganese	1.63	1.36
Iron	81.89	15.46
Cobalt	<MDL	0.02
Copper	4.98	3.06
Zinc	134.62	72.11
Arsenic	0.11	<MDL
Selenium	0.84	<MDL
Cadmium	<MDL	<MDL
Mercury	0.12	0.13
Lead	0.06	<MDL
Uranium	<MDL	<MDL
Chromium	0.42	<MDL

*Reliance on retrospective review of medical records for clinical parameters resulted in unknown values for measurements not studied during clinical encounter.

## Discussion

Several hypotheses on the etiology of MeN exist, most notably: excessive or repeated heat stress coupled with dehydration, exposure to toxic agrochemicals, overuse of nephrotoxic medications, homemade alcohol consumption, traditional/healing herbs, infection, and exposure to nephrotoxic heavy metals. No one hypothesis is yet sufficiently substantiated. We previously surmised that the clinical and epidemiologic characteristics of MeN suggest an underlying origin involving, at least in part, exposure to an exogenous, pro-inflammatory agent, most likely from an environmental source [12–14].

This study documents nephrotoxic trace element exposures among agricultural workers in Nicaragua and provides the first evidence of differential exposure among individuals with renal injury compared to individuals with healthy renal function. These data suggest that a specific element, or combination of elements, likely contributes to renal injury in a setting where MeN is hyperendemic. Specifically, higher concentrations of Ni were observed among renal patients in this study than in healthy controls from the same population, and an inverse dose-response relationship was observed between Ni concentrations and renal filtration. Although Ni exposure, mostly low-level, was also evident in some healthy subjects, this study provides new, compelling evidence that Ni, even at low doses, is associated with increased risk of renal injury, systemic inflammation, and anemia–hallmarks of acute MeN.

Several trace elements were detected in this study for which toxic levels have known adverse health effects, some at higher concentrations than reference values from U.S. and Canadian populations [19, 21]. In our study, Ni levels exceeded reference ranges in renal patient toenails, while controls had levels within normal limits; higher concentrations were also noted for Al, V, Mn, Fe, Cu, As, and Pb. Since V exposure was more common to renal patients, its role in MeN or other renal insults should also be vetted. The low levels of Se we observed may have health implications, renal or otherwise, since it is largely considered an essential element that protects against heavy metal toxicity. Although the effects of mixtures should still be considered, specific potentially nephrotoxic elements, such as U, As, Cd, Hg, and Pb might be ruled out at this stage.

Drastic drops in creatinine clearance among renal patients was accompanied by marked decline in hematocrit levels, despite normal measures in the same subjects only months earlier. Thus, we observed acute anemia among acute renal patients who were not previously anemic. In prior reports, 60% of acute MeN were anemic, and anemia predicted CKD progression [12, 13]. Although anemia is often a consequence of renal damage, the acute nature of renal injury, normal baseline measurements just months prior during the annual screening visit, and lack of evidence of CKD in our case-patients make it unlikely that the anemia developed over a substantial period of time. These data reiterate our previous observation of the acute nature of the anemia [13]. We suppose that an etiologic agent of MeN could injure the kidneys and induce an anemic state observed both in the early phase of MeN and during MeN CKD [13, 16].

Ni intoxication typically presents as nausea, vomiting, headaches, weakness, and dizziness, often resembling a mild, flu-like illness [22]. Major consequences of Ni intoxication remain poorly described, but animal studies reflect anemia, injury to kidneys or liver, immune and biochemical dysregulation, and increased mortality; in utero exposures were associated with reduced growth and development, including small kidneys and impaired development of immune and other important processes [22–28]. Physiologic damage primarily involves generation of harmful reactive oxygen species, modifications to gene expressions, and modulation or inhibition of metabolic pathways. Ni also causes direct damage to DNA, inhibits repair systems, and prevents methylation. It injures tissues through cytotoxicity, indirect damage (notably, to renal tubular epithelial cells), and immune dysregulation, processes known to accelerate renal disease and increase risk of ESRD through chronic exposures and repeat injury [29–31]. Still, surprisingly little is known about long-term effects of Ni on human kidneys or health status, and evidence regarding low-dose, repeat, or chronic exposure or in combination with other exposures, is lacking.

The kidney accumulates excess metal ions through reabsorption, and in the setting of repeat or chronic exposures, even when the exposure level is low, the potential for renal injury is considerable [32]. Once damaged, increased susceptibility to further insult can accelerate loss of renal mass and function, leading to rapidly progressing and severe disease [31, 33]. In particular, the kidney is the primary target organ for Ni accumulation, and a link between Ni exposure and end-stage renal disease has already been described [25, 31]. Ni exposed renal tissue reveals tubular injury with interstitial inflammation and focal damage concentrated at the corticomedullary junction [24, 25] which is consistent with our findings on biopsy of acute MeN cases [14].

Clinical observations of acute MeN bear remarkable similarity to those of Ni intoxication–namely, flu-like illness with leukocytosis, neutrophilia, lymphopenia, and anemia, accompanied by tubular interstitial nephritis with immune cell infiltrate focused about the corticomedullary junction [12–14, 22, 34]. The higher risk of Ni exposure with younger age in this study is also consistent with the young age at which acute (median 28 years) and chronic (median 32 years) MeN is observed [13]. Since we leveraged an occupational cohort for this investigation, we did not assess exposure or disease in children or adolescents. There is a real need for studies in children and also women, since consequences of fetal Ni exposure may impact susceptibility to MeN [22]. The clinical and epidemiologic parallels between Ni intoxication and the early stages of MeN gleaned from this study and others may point to Ni as an important and unrecognized cause of kidney disease in this region, and exposure effects should be thoroughly investigated in the pathway to MeN.

Ni is an abundant, naturally occurring element. Chronic or recurrent exposure to Ni can arise from either anthropogenic or geogenic sources, and most exposures occur through incidental ingestion, via Ni-enriched water (e.g. surface or ground water), food, or soil. Few exposure mechanisms could explain a widespread, yet geographically mosaic, environmental distribution. Unique geomorphologic, hydrogeologic, and topographic features of the Pacific region of Nicaragua and neighboring countries include mantle rocks and magma pockets rich in certain elements, including Ni [35–37]. Moved by mantle shifts, over time, Ni and Ni-bearing minerals in the region have been pushed closer to the earth’s surface. Through both natural means (e.g. weathering, erosion, and volcanic activity) and human activities (e.g. mining, well-digging, and irrigation), mineral sediments can be repositioned into the human environment. Minerals could reasonably be expected to leach into drinking water, creating an broad exposure source in the nearby communities [22]. Natural depositional processes, such as air and water flow, can act to concentrate sediments in certain locales, where individuals with heavy or repeated exposures to Ni-rich soil and water would have higher doses and/or frequencies than elsewhere or than individuals without such contact. Agricultural field laborers, miners, and brick-makers have the highest risk of MeN [11]. Importantly, while evidence exists that MeN is more frequent among certain occupational activities, no occupational exposure source has yet been identified. Herein, we suggest that the exposure source must be widely distributed, and exposure intensity and frequency depend on various factors (e.g. geography, contact type, water source, water consumption). If Ni exposure is involved in the genesis of MeN, a geologic source is the most plausible origin. The unique mineralogic features of the region, likely dispersal patterns that overlay with MeN regions, and clinico-epidemiologic evidence support the plausibility of this hypothesis [35, 36, 38].

There are several limitations to our study that are worth discussing. Although we made every effort to correctly classify cases and controls, unknown renal events are a possibility that could influence case or control misclassification. Likewise, with a lack of consensus on the case definition of MeN in the early stages, we cannot be absolutely certain that all renal cases in our study are definitely MeN, nor that we can extrapolate our data to asymptomatic or mild MeN cases who do not seek care. However, the clinical presentation of case-patients in this analysis are consistent with those in prior studies, including cases who later developed MeN CKD. Follow-up of this cohort will confirm if additional subjects develop CKD attributed to MeN and confirm if Ni exposure contributes to disease or its progression. Toenail from one control participant reflected high Ni concentration, yet no acute nor historical renal impairment was noted; it is unknown if any controls had undetected renal events or endured exposure without clinical consequence. It is important to consider that even exposed individuals may not be susceptible to injury or may have only mild or subclinical responses. Similarly, low dose, infrequent, or brief exposures may not elicit physiologic response in all individuals. It will be important to understand more about underlying host susceptibility factors (e.g. genetic, immune, nutritional, hydration status, etc), including gene-environmental interactions, that could be involved in the initiation and progression of MeN.

Validated biomarkers of Ni exposure and of MeN do not yet exist. Toenail concentrations reflect accumulated exposure, though metabolism and accumulation may be influenced by unmeasured variables. As such, nail concentrations do not perfectly reflect exposures within a discreet, known window of time, and thus we did not directly measure element concentrations at the exact, unknown moment of exposure. To detect trace minerals in toenails, it is important to collect specimens at least 3 months following suspected exposure to allow for growth of the nail so that detection is optimized. Other factors (e.g. exposure dose or duration, growth rate of individuals’ nails, mass of nails collected) could affect our use of a 3-month post-exposure measurement in capturing very recent, brief-acute or longer-term exposures. While these case-patients had normal baseline renal function and lacked a history of long-standing renal disease, we cannot completely rule out reverse causation in this cohort, with Ni accumulation occurring secondary to AKI as a result of decreased GFR. To help rule out reverse causation, future studies should include controls from the same geographic region with AKI or CKD confirmed to be related to other causes. Nevertheless, existing data on in nickel clearance help support our findings [39]. Finally, detection of certain elements may be limited in specimens of low mass (i.e. the probability of detection decreases in lower mass nail clippings), and many specimens in our study were small (although not differentially so between cases and controls). Despite these potential limitations, nails are a useful and validated tool for fingerprinting occupational and other environmental exposures to trace elements and assessing the exposure dose, and our methods are expected to reliably capture exposures, even low doses, over a prior 3–12 month period [34, 40–42]. Our use of toenails as a marker of exposure history in a closely followed, well-documented cohort of acute MeN is an important strength of this study, and this analysis provides important new insight into exposures using the most diverse panel of trace elements so far in the study of MeN.

Achieving additional insight into the underlying cause of MeN is an enormous milestone in curbing the epidemic. At this stage, further exposure analyses and environmental sampling in the community-at-large are urgently needed to evaluate the role of Ni and other elements in disease. There is an immediate need to (1) measure trace-element toxicity in a large sample of individuals with acute MeN in order to ensure adequate sample size and power, (2) determine if areas with high concentrations in the environment to which people may be exposed correspond to known MeN hotspots, and (3) confirm the exposure source and pathway to better understand risk and devise public health interventions. Ongoing investigation and analysis of environmental specimens and geochemical processes across this region will confirm the origin, geographic reach, environmental transmission dynamics, and exposure pathway of the toxicant, informing remediation and prevention efforts. It will also be important to understand epigenetic consequences of exposure, assess effects of environmental mixtures and interactions, and determine if exposures during childhood drive this disease in young adulthood. Fortunately, strategies to treat and prevent metal intoxications are largely accessible; if Ni toxicity is proved to be a catalyst of MeN, treatment algorithms (e.g. chelation) and exposure-reducing interventions (e.g. public health measures, environmental remediation, or clean water provision in heavy labor settings) for high-risk communities, can be rapidly implemented and monitored. Interrupting the pathway to acute renal toxicity and thereby reducing the high renal morbidity and mortality in this region will curb this decades-long epidemic mystery, improving health and well-being across Mesoamerica.

## Supporting information

S1 Fig Relationship between toenail Aluminum concentrations (log10-mg/kg dry nail mass) and select physiologic parameters. (DOCX)

S2 Fig Relationship between toenail Vanadium concentrations (log10-mg/kg dry nail mass) and select physiologic parameters. (DOCX)

S1 Table Toenail trace element concentrations (mg/kg dry nail mass) of renal patients and healthy controls, compared to healthy reference populations (n = 54). (DOCX)

S2 Table Heavy metals detected in toenails of renal patients and healthy controls (n = 54). (DOCX)

S3 Table Acute clinical presentation. (DOCX)

S4 Table Correlations between trace elements toenail concentrations (mg/kg dry nail mass) (n = 54). (DOCX)
