# Supplementary material for: Evidence of nickel and other trace elements and their relationship to clinical findings in acute Mesoamerican Nephropathy: A case-control analysis
Source: PLoS One. 2020 Nov 10;15(11):e0240988. doi: 10.1371/journal.pone.0240988 (PMC7654766; doi:10.1371/journal.pone.0240988)
Supplement: S1 Fig — (DOCX) [file pone.0240988.s001.docx]

**S1 Fig.** Relationship between toenail Aluminum concentrations (log10-mg/kg dry nail mass) and select physiologic parameters

β= 0.206

p=0.161

n=48

β= -0.096

p=0.490

n=54

β= 0.322

p=0.026*

n=48

β= -0.194

p=0.279

n=33

β= -0.131

p=0.356

n=52

β= -0.219

p= 0.135

n=48

β= -0.292

p=0.110

n=31

β= 0.049

p=0.744

n=47

β= -0.308

p=0.037*

n=46

β= 0.291

p=0.045*

n=48

β= -0.218

p=0.151

n=45

β= -0.302

p=0.027*

n=54

*Statistically significant at p<0.05
