# Supplementary material for: Evidence of nickel and other trace elements and their relationship to clinical findings in acute Mesoamerican Nephropathy: A case-control analysis
Source: PLoS One. 2020 Nov 10;15(11):e0240988. doi: 10.1371/journal.pone.0240988 (PMC7654766; doi:10.1371/journal.pone.0240988)
Supplement: S2 Fig — (DOCX) [file pone.0240988.s002.docx]

**S2 Fig.** Relationship between toenail Vanadium concentrations (log10-mg/kg dry nail mass) and select physiologic parameters

β= 0.305

p=0.205

n=19

β= -0.070

p=0.754

n=22

β= 0.418

p=0.075

n=19

β= -0.122

p=0.690

n=13

β= -0.193

p=0.389

n=22

β= -0.318

p= 0.185

n=19

β= -0.536

p=0.059

n=13

β= 0.190

p=0.421

n=20

β= -0.458

p=0.056

n=18

β= -0.039

p=0.877

n=88

β= -0.412

p=0.056

n=22

β= 0.477

p=0.053

n=17

*Statistically significant at p<0.05
