# Supplementary material for: Evidence of nickel and other trace elements and their relationship to clinical findings in acute Mesoamerican Nephropathy: A case-control analysis
Source: PLoS One. 2020 Nov 10;15(11):e0240988. doi: 10.1371/journal.pone.0240988 (PMC7654766; doi:10.1371/journal.pone.0240988)
Supplement: S1 Table — (DOCX) [file pone.0240988.s003.docx]

| **S1 Table. Toenail trace element concentrations (mg/kg dry nail mass) of Renal Patients and Healthy Controls, compared to Healthy Reference Populations (n=54)** | | | | | | | | | | | | | | | | |
| --- | --- | --- | --- | --- | --- | --- | --- | --- | --- | --- | --- | --- | --- | --- | --- | --- |
|  |  | **Control** | | **Case** | ALL | | | Χ^2^ | **No AKI** | | | **AKI** | ALL | | Χ^2^ | |
|  |  | 36 (66.7%) | | 18 (33.3%) | 54 (100%) | | | p-value | 32 (66.7%) | | | 16 (33.3%) | 48 (100%) | | p-value | |
| **Concentration of element Outside Reference Range** | | | | | | | | | | | | | | | | |
| Nickel >0.79 mg/kg | ^¥^ | 5 (23.8%) | | 14 (77.8%) | 19 (48.7%) | | | 0.001* | 4 (21.1%) | | | 14 (87.5%) | 18 (51.4%) | | <0.001* | |
| Nickel >2.48 mg/kg | ^≠^ | 1 (4.8%) | | 8 (44.4%) | 9 (23.1%) | | | 0.006* | 1 (5.3%) | | | 8 (50%) | 9 (25.7%) | | 0.005* | |
| Aluminum >8.09 mg/kg | ^¥^ | 35 (97.2%) | | 17 (94.4%) | 52 (96.3%) | | | 1.000 | 32 (100%) | | | 15 (93.8%) | 47 (97.9%) | | 0.333 | |
| Aluminum >22.97 mg/kg | ^≠^ | 28 (77.8%) | | 17 (94.4%) | 45 (83.3%) | | | 0.244 | 26 (81.3%) | | | 15 (93.8%) | 41 (85.4%) | | 0.398 | |
| Vanadium >0.030 mg/kg | ^£^ | 18 (100%) | | 4 (100%) | 22 (100%) | | | n/a | 14 (100%) | | | 3 (100%) | 17 (100%) | | n/a | |
| Vanadium >0.07 mg/kg | ^€^ | 16 (88.9%) | | 4 (100%) | 20 (90.9%) | | | 1.000 | 13 (92.9%) | | | 3 (100%) | 16 (94.1%) | | 1.000 | |
| Manganese >0.31 mg/kg | ^¥^ | 30 (93.8%) | | 17 (100%) | 47 (95.9%) | | | 0.537 | 29 (93.6%) | | | 15 (100%) | 44 (95.7%) | | 1.000 | |
| Manganese >1.01 mg/kg | ^≠^ | 24 (75%) | | 16 (94.1%) | 40 (81.6%) | | | 0.136 | 23 (74.2%) | | | 14 (93.3%) | 37 (80.4%) | | 0.235 | |
| Iron >12.21 mg/kg | ^¥^ | 30 (88.2%) | | 16 (94.1%) | 46 (90.2%) | | | 0.654 | 26 (86.7%) | | | 14 (93.3%) | 40 (88.9%) | | 0.651 | |
| Iron >26.74 mg/kg | ^≠^ | 23 (67.7%) | | 14 (82.4%) | 37 (72.6%) | | | 0.334 | 20 (66.7%) | | | 12 (80%) | 32 (71.1%) | | 0.492 | |
| Cobalt >0.02 mg/kg | ^¥^ | 0 (0%) | | 5 (71.4%) | 5 (50%) | | | 1.000 | 2 (66.7%) | | | 6 (85.7%) | 8 (80%) | | 1.000 | |
| Cobalt >0.05 mg/kg | ^≠^ | 1 (33.3%) | | 6 (85.7%) | 7 (70%) | | | 0.183 | 1 (33.3%) | | | 6 (85.7%) | 7 (70%) | | 0.183 | |
| Copper >3.46 mg/kg | ^¥^ | 21 (58.3%) | | 14 (77.8%) | 35 (64.8%) | | | 0.229 | 21 (65.6%) | | | 12 (75%) | 33 (68.8%) | | 0.742 | |
| Copper >5.40 mg/kg | ^≠^ | 7 (19.4%) | | 5 (27.8%) | 12 (22.2%) | | | 0.506 | 6 (18.8%) | | | 5 (31.3%) | 11 (22.9%) | | 0.468 | |
| Zinc >108.49 mg/kg | ^¥^ | 10 (27.8%) | | 2 (11.1%) | 12 (22.2%) | | | 0.298 | 7 (21.9%) | | | 2 (12.5%) | 9 (18.8%) | | 0.697 | |
| Arsenic >0.05 mg/kg | ^¥^ | 14 (93.3%) | | 5 (83.3%) | 19 (90.5%) | | | 0.500 | 13 (92.9%) | | | 4 (80%) | 17 (89.5%) | | 0.468 | |
| Arsenic >0.13 mg/kg | ^≠^ | 10 (66.7%) | | 3 (50%) | 13 (61.9%) | | | 0.631 | 8 (57.1%) | | | 3 (60%) | 11 (57.9%) | | 1.000 | |
| Selenium <0.74 mg/kg | ^©^ | 11 (57.9%) | | 1 (25%) | 12 (52.2%) | | | 0.317 | 9 (56.3%) | | | 1 (33.3%) | 10 (52.6%) | | 0.582 | |
| Selenium <0.93 mg/kg | ^¥^ | 15 (79%) | | 3 (75%) | 18 (78.3%) | | | 1.000 | 14 (87.5%) | | | 2 (66.7%) | 16 (84.2%) | | 0.422 | |
| Cadmium >0.02 mg/kg | ^¥^ | 5 (38.5%) | | 5 (62.5%) | 10 (47.6%) | | | 0.387 | 4 (33.3%) | | | 4 (57.1%) | 8 (42.1%) | | 0.377 | |
| Cadmium >0.35 mg/kg | ^≠^ | 0 (0%) | | 0 (0%) | 0 (0%) | | | n/a | 0 (0%) | | | 0 (0%) | 0 (0%) | | n/a | |
| Mercury >0.38 mg/kg | ^£^ | 0 (0%) | | 1 (9.1%) | 1 (3.2%) | | | 0.355 | 0 (0%) | | | 1 (10%) | 1 (3.6%) | | 0.357 | |
| Lead >0.04 mg/kg | ^¥^ | 15 (88.2%) | | 4 (100%) | 19 (90.5%) | | | 1.000 | 11 (84.6%) | | | 3 (100%) | 14 (87.5%) | | 1.000 | |
| Lead >0.15 mg/kg | ^≠^ | 5 (29.4%) | | 1 (25%) | 6 (28.6%) | | | 1.000 | 2 (15.4%) | | | 1 (33.3%) | 3 (18.8%) | | 0.489 | |
| Uranium >0.01 mg/kg | ^≠^ | 1 (20%) | | 2 (66.7%) | 3 (37.5%) | | | 0.464 | 1 (25%) | | | 2 (66.7%) | 3 (42.9%) | | 0.486 | |
| Chromium >1.13 mg/kg | ^¥^ | 2 (25%) | | 2 (40%) | 4 (30.8%) | | | 1.000 | 2 (25%) | | | 2 (66.7%) | 4 (36.4%) | | 0.491 | |
| *Statistically significant at p<0.05 | | |  | | |  |  | |  |  |  | | |  | |  |
| ^≠^90th percentile concentration (Unrine et al., 2019) | | | | | |  |  | |  |  |  | | |  | |  |
| ^≠^90th percentile concentration (Unrine et al., 2019) | | | | | |  |  | |  |  |  | | |  | |  |
| ^≠^90th percentile concentration (Unrine et al., 2019) | | | | | |  |  | |  |  |  | | |  | |  |
| ^£^50th percentile concentration (Goullé et al., 2009) | | |  | | |  |  | |  |  |  | | |  | |  |
| ^€^95th percentile concentration (Goullé et al., 2009) | | |  | | |  |  | |  |  |  | | |  | |  |
