# Supplementary material for: Evidence of nickel and other trace elements and their relationship to clinical findings in acute Mesoamerican Nephropathy: A case-control analysis
Source: PLoS One. 2020 Nov 10;15(11):e0240988. doi: 10.1371/journal.pone.0240988 (PMC7654766; doi:10.1371/journal.pone.0240988)
Supplement: S2 Table — (DOCX) [file pone.0240988.s004.docx]

| **S2 Table. Heavy Metals Detected in Toenails of Renal Patients and Healthy Controls (n=54)** | | | | | | | | | | | |
| --- | --- | --- | --- | --- | --- | --- | --- | --- | --- | --- | --- |
| **Method Detection Limit; MDL (mg/kg dry nail mass)**; median (range) | | | | | | | | | | | |
|  | **Control** | | **Case** | | ALL | | Χ^2^ | **No AKI** | **AKI** | ALL | Χ^2^ |
|  | 36 (66.7%) | | 18 (33.3%) | | 54 (100%) | | p-value | 32 (66.7%) | 16 (33.3%) | 48 (100%) | p-value |
| Ni | 0.09 (0.01, 2.75) | | 0.12 (0.03, 1.10) | | 0.11 (0.03, 2.75) | | 0.650 | 0.12 (0.01, 2.75) | 0.12 (0.03, 1.10) | 0.12 (0.01, 2.75) | 0.956 |
| Al | 1.34 (0.13, 39.77) | | 1.78 (0.56, 15.91) | | 1.65 (0.13, 39.77) | | 0.645 | 1.73 (0.13, 39.77) | 1.78 (0.57, 15.91) | 1.78 (0.13, 39.77) | 0.955 |
| V | 0.02 (0.003, 0.63) | | 0.03 (0.01, 0.25) | | 0.03 (0.003, 0.6) | | 0.767 | 0.03 (0.003, 0.63) | 0.03 (0.01, 0.25) | 0.03 (0.003, 0.63) | 0.228 |
| Mn | 0.19 (0.02, 3.61) | | 0.07 (0.02, 0.38) | | 0.15 (0.02, 3.61) | | 0.162 | 0.17 (0.02, 3.61) | 0.07 (0.02, 0.38) | 0.13 (0.01, 3.61) | 0.000 |
| Fe | 2.71 (0.34, 56.3) | | 2.51 (0.78, 22.51) | | 2.64 (0.34, 56.27) | | 0.917 | 3.00 (0.77, 56.27) | 2.51 (0.79, 22.52) | 2.76 (0.77, 56.27) | 0.794 |
| Co | 0.12 (0.01, 2.20) | | 0.02 (0.01, 0.17) | | 0.07 (0.01, 2.20) | | 0.049* | 0.11 (0.005, 2.20) | 0.02 (0.01, 0.17) | 0.04 (0.01, 2.20) | 0.043* |
| Cu | 0.05 (0.01, 1.39) | | 0.02 (0.01, 0.13) | | 0.04 (0.01, 1.39) | | 0.342 | 0.05 (0.005, 1.39) | 0.02 (0.01, 0.13) | 0.04 (0.01, 1.39) | 0.417 |
| Zn | 0.37 (0.02, 12.16) | | 0.54 (0.06, 4.87) | | 0.50 (0.02, 12.16) | | 0.621 | 0.53 (0.02, 12.16) | 0.54 (0.07, 4.87) | 0.54 (0.02, 12.16) | 0.953 |
| As | 0.07 (0.01, 0.95) | | 0.04 (0.02, 0.38) | | 0.05 (0.01, 0.95) | | 0.151 | 0.06 (0.02, 0.95) | 0.04 (0.02, 0.38) | 0.05 (0.02, 0.95) | 0.335 |
| Se | 0.29 (0.034, 8.04) | | 0.36 (0.08, 3.21) | | 0.33 (0.03, 8.04) | | 0.703 | 0.35 (0.03, 8.04) | 0.36 (0.08, 3.21) | 0.36 (0.03, 8.04) | 0.955 |
| Cd | 0.01 (0.002, 0.18) | | 0.01 (0.003, 0.07) | | 0.01 (0.002, 0.18) | | 0.895 | 0.01 (0.002, 0.18) | 0.01 (0.003, 0.07) | 0.01 (0.002, 0.18) | 0.626 |
| Hg | 0.08 (0.01, 1.75) | | 0.08 (0.03, 0.70) | | 0.08 (0.01, 1.75) | | 0.941 | 0.09 (0.01, 1.75) | 0.08 (0.03, 0.70) | 0.09 (0.01, 1.75) | 0.789 |
| Pb | 0.04 (0.01, 1.09) | | 0.05 (0.01, 0.44) | | 0.05 (0.01, 1.09) | | 0.767 | 0.05 (0.01, 1.09) | 0.05 (0.01, 0.44) | 0.05 (0.01, 1.09) | 0.857 |
| U | 0.01 (0.002, 0.18) | | 0.01 (0.003, 0.07) | | 0.01 (0.002, 0.18) | | 0.937 | 0.01 (0.002, 0.18) | 0.01 (0.003, 0.07) | 0.01 (0.002, 0.18) | 0.703 |
| Cr | 0.19 (0.02, 5.98) | | 0.27 (0.04, 2.39) | | 0.25 (0.02, 5.98) | | 0.639 | 0.26 (0.02, 5.98) | 0.27 (0.04, 2.39) | 0.27 (0.02, 5.98) | 0.955 |
| **Proportion of Specimens Measured at Concentration ≥MDL**; n (%) | | | | | | | | | | | |
|  | | **Control** | | **Case** | ALL | | Χ^2^ | **No AKI** | **AKI** | ALL | Χ^2^ |
|  | | 36 (66.7%) | | 18 (33.3%) | 54 (100%) | | p-value | 32 (66.7%) | 16 (33.3%) | 48 (100%) | p-value |
| Ni | | 21 (58.3%) | | 18 (100%) | 39 (72.2%) | | 0.001* | 19 (59.4%) | 16 (100%) | 35 (72.9%) | 0.002* |
| Al | | 36 (100%) | | 18 (100%) | 54 (100%) | | n/a | 32 (100%) | 16 (100%) | 48 (100%) | n/a |
| V | | 18 (50.0%) | | 4 (22.2%) | 22 (40.7%) | | 0.078 | 14 (43.8%) | 3 (18.8%) | 17 (35.4%) | 0.116 |
| Mn | | 32 (88.9%) | | 17 (94.4%) | 49 (90.7%) | | 0.655 | 31 (96.9%) | 15 (93.8%) | 46 (95.8%) | 1.000 |
| Fe | | 34 (94.4%) | | 17 (94.4%) | 51 (94.4%) | | 1.000 | 30 (93.8%) | 15 (93.8%) | 45 (93.8%) | 1.000 |
| Co | | 3 (8.3%) | | 7 (38.9%) | 10 (18.5%) | | 0.011* | 3 (9.4%) | 7 (43.8%) | 10 (20.8%) | 0.010* |
| Cu | | 36 (100%) | | 18 (100%) | 54 (100%) | | n/a | 32 (100%) | 16 (100%) | 48 (100%) | n/a |
| Zn | | 36 (100%) | | 18 (100%) | 54 (100%) | | n/a | 32 (100%) | 16 (100%) | 48 (100%) | n/a |
| As | | 15 (41.7%) | | 6 (33.3%) | 21 (38.9%) | | 0.554 | 14 (43.8%) | 5 (31.3%) | 19 (39.6%) | 0.535 |
| Se | | 19 (52.8%) | | 4 (22.2%) | 23 (42.6%) | | 0.043* | 16 (50.0%) | 3 (18.8%) | 19 (39.6%) | 0.060 |
| Cd | | 13 (36.1%) | | 8 (44.4%) | 21 (38.9%) | | 0.554 | 12 (37.5%) | 7 (43.8%) | 19 (39.6%) | 0.759 |
| Hg | | 20 (55.6%) | | 11 (61.1%) | 31 (57.4%) | | 0.697 | 18 (56.3%) | 10 (62.5%) | 28 (58.3%) | 0.679 |
| Pb | | 17 (47.2%) | | 4 (22.2%) | 21 (38.9%) | | 0.138 | 13 (40.6%) | 3 (18.8%) | 16 (33.3%) | 0.196 |
| U | | 5 (13.9%) | | 3 (16.7%) | 8 (14.8%) | | 1.000 | 4 (12.5%) | 3 (18.8%) | 7 (14.6%) | 0.672 |
| Cr | | 8 (22.2%) | | 5 (27.8%) | 13 (24.1%) | | 0.740 | 8 (25.0%) | 3 (18.8%) | 11 (22.9%) | 0.729 |
| **Proportion of Specimens Measured at Concentrations ≥50th Percentile;** ; n(%) | | | | | | | | | | | |
| Mn ≥2.32 mg/kg | | 14 (43.8%) | | 10 (58.8%) | | 24 (49%) | 0.315 | 13 (41.9%) | 9 (60%) | 22 (47.8%) | 0.250 |
| Fe ≥61.22 mg/kg | | 15 (44.1%) | | 10 (58.8%) | | 25 (49%) | 0.322 | 13 (43.3%) | 9 (60%) | 22 (48.9%) | 0.292 |
| Co ≥0.10 mg/kg | | 0 (0%) | | 5 (71.4%) | | 5 (50%) | 0.167 | 0 (0%) | 5 (71.4%) | 5 (50%) | 0.167 |
| Cu ≥3.91 mg/kg | | 16 (44.4%) | | 11 (61.1%) | | 27 (50%) | 0.248 | 17 (53.1%) | 9 (56.3%) | 26 (54.2%) | 0.838 |
| Zn ≥98.78 mg/kg | | 18 (50.0%) | | 9 (50.0%) | | 27 (50%) | 1.000 | 17 (53.1%) | 7 (43.8%) | 24 (50.0%) | 0.540 |
| As ≥0.16 mg/kg | | 8 (53.3%) | | 2 (33.3%) | | 10 (47.6%) | 0.635 | 6 (42.9%) | 2 (40.0%) | 8 (42.1%) | 1.000 |
| Se ≥0.73 mg/kg | | 8 (42.1%) | | 3 (75%) | | 11 (47.8%) | 0.317 | 7 (43.8%) | 2 (66.7%) | 9 (47.4%) | 0.582 |
| Cd ≥0.02 mg/kg | | 6 (46.2%) | | 5 (62.5%) | | 11 (52.4%) | 0.659 | 5 (41.7%) | 4 (57.1%) | 9 (47.4%) | 0.650 |
| Hg ≥0.12 mg/kg | | 8 (40.0%) | | 7 (63.6%) | | 15 (48.4%) | 0.273 | 8 (44.4%) | 7 (70%) | 15 (53.6%) | 0.254 |
| Pb ≥0.07 mg/kg | | 8 (47.1%) | | 3 (75.0%) | | 11 (52.4%) | 0.586 | 5 (38.5%) | 3 (100%) | 8 (50.0%) | 0.200 |
| *Statistically significant at p<0.05 | | | | | | | | | | | |
