# Supplementary material for: Evidence of nickel and other trace elements and their relationship to clinical findings in acute Mesoamerican Nephropathy: A case-control analysis
Source: PLoS One. 2020 Nov 10;15(11):e0240988. doi: 10.1371/journal.pone.0240988 (PMC7654766; doi:10.1371/journal.pone.0240988)
Supplement: S3 Table — (DOCX) [file pone.0240988.s005.docx]

| **S3 Table. Acute clinical presentation** | | | |  |  |  |  |  |  |  |  |  |  |  |  |
| --- | --- | --- | --- | --- | --- | --- | --- | --- | --- | --- | --- | --- | --- | --- | --- |
|  |  |  | **Control** | **Case** | **ALL** |  | **Nickel <MDL** | **Nickel ≥MDL** |  | **Nickel ≤0.79mg/kg** | **Nickel >0.79mg/kg** |  | **Nickel ≤2.48mg/kg** | **Nickel >2.48mg/kg** |  |
|  |  |  | 36 (66.7%) | 18 (33.3%) |  | p-value | 15 (27.8%) | 39 (72.2%) | p-value | 20 (51.3%) | 19 (48.7%) | p-value | 30 (76.9%) | 9 (23.1%) | p-value |
| **Medical History*** | | |  |  |  |  |  |  |  |  |  |  |  |  |  |
|  | Prior Elevated Creatinine | | 3 (8.3%) | 5 (29.4%) | 8 (15.1%) | 0.094 | 0 (0%) | 8 (21.1%) | 0.088 | 6 (30.0%) | 2 (11.1%) | 0.238 | 7 (24.14%) | 1 (11.1%) | 0.650 |
|  | Hypertension | | 1 (2.8%) | 2 (11.8%) | 3 (5.7%) | 0.238 | 0 (0%) | 3 (7.9%) | 0.550 | 2 (10.0%) | 1 (5.6%) | 1.000 | 3 (10.3%) | 0 (0%) | 1.000 |
|  | Anemia | | 4 (11.1%) | 2 (11.8%) | 6 (11.3%) | 1.000 | 1 (6.7%) | 5 (13.2%) | 0.662 | 4 (20.0%) | 1 (5.6%) | 0.344 | 4 (13.8%) | 1 (11.1%) | 1.000 |
|  | Malaria | | 10 (27.8%) | 4 (23.5%) | 14 (26.4%) | 1.000 | 4 (26.7%) | 10 (26.3%) | 1.000 | 8 (40.0%) | 2 (11.1%) | 0.067 | 8 (27.59%) | 2 (22.2%) | 1.000 |
|  | Dengue | | 0 (0%) | 3 (17.7%) | 3 (5.8%) | 0.031 | 0 (0%) | 3 (7.9%) | 0.555 | 2 (10.0%) | 1 (5.6%) | 1.000 | 2 (6.9%) | 1 (11.1%) | 1.000 |
| **Parental History of CKD^¥^** | | | 8 (25.8%) | 3 (60%) | 11 (30.6%) | 0.154 | 5 (45.5%) | 10 (40.0%) | 1.000 | 7 (41.2%) | 3 (37.5%) | 1.000 | 8 (36.4%) | 2 (66.7%) | 0.543 |
| **Symptoms^±^** | | |  |  |  |  |  |  |  |  |  |  |  |  |  |
|  | Fever/Chills | | 3 (8.3%) | 13 (76.5%) | 16 (30.2%) | <0.001 | 1 (6.7%) | 15 (39.5%) | 0.022 | 5 (25.0%) | 10 (55.6%) | 0.096 | 9 (31%) | 6 (66.7%) | 0.115 |
|  | N/V |  | 0 (0%) | 13 (72.2%) | 13 (24.1%) | <0.001 | 0 (0%) | 13 (33.3%) | 0.011 | 3 (15.0%) | 10 (52.6%) | 0.019 | 7 (23.3%) | 6 (66.7%) | 0.039 |
|  | Back Pain | | 5 (13.9%) | 12 (70.6%) | 17 (32.1%) | <0.001 | 1 (6.7%) | 16 (42.1%) | 0.020 | 6 (30.0%) | 10 (55.6%) | 0.188 | 10 (33.3%) | 6 (75.0%) | 0.050 |
|  | Headache | | 4 (11.4%) | 9 (52.9%) | 13 (25.0%) | 0.002 | 1 (6.7%) | 12 (32.4%) | 0.078 | 5 (26.3%) | 7 (38.9%) | 0.495 | 10 (34.5%) | 2 (25.0%) | 1.000 |
|  | Debility | | 2 (5.6%) | 5 (31.3%) | 7 (13.5%) | 0.023 | 1 (6.7%) | 6 (16.2%) | 0.658 | 2 (10.0%) | 4 (23.5%) | 0.383 | 4 (13.8%) | 2 (25.0%) | 0.591 |
|  | Paresthesia | | 0 (0%) | 4 (26.7%) | 4 (8.0%) | 0.006 | 0 (0%) | 4 (11.1%) | 0.566 | 1 (5.0%) | 3 (18.8%) | 0.303 | 3 (10.7%) | 1 (12.5%) | 1.000 |
|  | Cramps | | 1 (2.8%) | 9 (56.3%) | 10 (19.2%) | <0.001 | 0 (0%) | 10 (27%) | 0.046 | 5 (25.0%) | 5 (29.4%) | 1.000 | 9 (30%) | 1 (14.3%) | 0.674 |
|  | Abdominal Pain | | 3 (8.3%) | 8 (57.1%) | 11 (22.0%) | 0.001 | 0 (0%) | 11 (31.4%) | 0.021 | 4 (20.0%) | 7 (46.7%) | 0.144 | 6 (21.4%) | 5 (71.4%) | 0.021 |
|  | Myalgia | | 2 (5.6%) | 5 (31.3%) | 7 (13.5%) | 0.023 | 1 (6.7%) | 6 (16.2%) | 0.658 | 2 (10.0%) | 4 (23.5%) | 0.383 | 5 (16.7%) | 1 (14.3%) | 1.000 |
|  | Arthralgia | | 3 (8.3%) | 7 (43.8%) | 10 (19.2%) | 0.006 | 0 (0%) | 10 (27%) | 0.046 | 6 (30.0%) | 4 (23.5%) | 0.725 | 9 (30%) | 1 (14.3%) | 0.647 |
|  | Urinary Symptoms | | 2 (5.6%) | 5 (31.3%) | 7 (13.5%) | 0.023 | 0 (0%) | 7 (18.9%) | 0.093 | 2 (10.0%) | 5 (29.4%) | 0.212 | 3 (10.7%) | 4 (44.4%) | My |
|  | Fatigue | | 2 (5.6%) | 1 (7.1%) | 3 (6%) | 1.000 | 0 (0%) | 3 (8.6%) | 0.545 | 2 (10.0%) | 1 (6.7%) | 1.000 | 2 (7.1%) | 1 (14.3%) | 0.499 |
|  | Neck Pain | | 2 (5.6%) | 1 (7.14%) | 3 (6%) | 1.000 | 0 (0%) | 3 (8.57%) | 0.545 | 3 (15.0%) | 0 (0%) | 0.244 | 3 (10.71%) | 0 (0%) | 1.000 |
|  | Cough | | 3 (8.3%) | 2 (13.3%) | 5 (9.8%) | 0.624 | 1 (6.7%) | 4 (11.1%) | 1.000 | 1 (5.0%) | 3 (18.8%) | 0.303 | 2 (6.9%) | 2 (28.6%) | 0.163 |
|  | Chest pain | | 1 (2.78%) | 1 (7.14%) | 2 (4%) | 0.486 | 0 (0%) | 2 (5.71%) | 1.000 | 1 (5.0%) | 1 (6.7%) | 1.000 | 2 (7.14%) | 0 (0%) | 1.000 |
|  | Blurred Vision | | 2 (5.56%) | 0 (0%) | 2 (4%) | 1.000 | 0 (0%) | 2 (5.71%) | 1.000 | 2 (10.0%) | 0 (0%) | 0.496 | 2 (7.14%) | 0 (0%) | 1.000 |
|  | Dizziness | | 1 (2.9%) | 6 (46.2%) | 7 (14.9%) | 0.001 | 0 (0%) | 7 (21.2%) | 0.086 | 4 (21.1%) | 3 (21.4%) | 1.000 | 6 (23.1%) | 1 (14.3%) | 1.000 |
|  | Respiratory Difficulty/Dyspnea | | 2 (5.6%) | 2 (14.3%) | 4 (8.0%) | 0.310 | 0 (0%) | 4 (11.4%) | 0.302 | 2 (10.0%) | 2 (13.3%) | 1.000 | 3 (10.7%) | 1 (14.3%) | 1.000 |
|  | Rash |  | 2 (5.6%) | 0 (0%) | 2 (4.0%) | 1.000 | 0 (0%) | 2 (5.71%) | 1.000 | 2 (10.0%) | 0 (0%) | 0.496 | 2 (7.1%) | 0 (0%) | 1.000 |
|  | Jaundice | | 1 (2.8%) | 0 (0%) | 1 (2.0%) | 1.000 | 1 (6.7%) | 0 (0%) | 0.300 | 0 (0%) | 0 (0%) | n/a | 1 (2.4%) | 0 (0%) | 1.000 |
| **Clinical Laboratory Parameters** | | | | |  |  |  |  |  |  |  |  |  |  |  |
|  | Creatinine >2.0 mg/dL | | 0 (0%) | 10 (55.6%) | 10 (18.5%) | <0.001 | 0 (0%) | 10 (25.6%) | 0.046 | 2 (10%) | 8 (42.1%) | 0.031 | 5 (16.7%) | 5 (55.6%) | 0.032 |
|  | Leukocytosis  (>10,000 cells/mm^3^) | | 1 (3.1%) | 15 (93.8%) | 16 (33.3%) | <0.001 | 0 (0%) | 16 (47.1%) | 0.002 | 2 (12.5%) | 14 (77.8%) | <0.001 | 8 (32%) | 8 (88.9%) | 0.006 |
|  | Moderate Leukocytosis  (>12,000 cells/mm^3^) | | 0 (0%) | 13 (81.3%) | 13 (27.1%) | <0.001 | 0 (0%) | 13 (38.2%) | 0.010 | 0 (0%) | 13 (72.2%) | <0.001 | 5 (20.0%) | 8 (88.9%) | 0.001 |
|  | Neutrophilia (>67%) | | 9 (28.1%) | 15 (93.8%) | 24 (50%) | <0.001 | 3 (21.4%) | 21 (61.8%) | 0.024 | 5 (31.3%) | 16 (88.9%) | 0.001 | 12 (.08%) | 9 (100%) | 0.006 |
|  | Lymphopenia (<21%) | | 1 (3.1%) | 9 (56.3%) | 10 (20.8%) | <0.001 | 0 (0%) | 10 (29.4%) | 0.023 | 1 (6.3%) | 9 (50%) | 0.008 | 4 (16.0%) | 6 (66.7%) | 0.009 |
|  | Eosinophilia (>4%) | | 0 (0%) | 0 (0%) | 0 (0%) | n/a | 0 (0%) | 0 (0%) | n/a | 0 (0%) | 0 (0%) | n/a | 0 (0%) | 0 (0%) | n/a |
|  | Hyperuricemia (mg/dL;  >7 male, >6 female) | | 4 (12.5%) | 6 (40%) | 10 (21.3%) | 0.054 | 2 (18.2%) | 8 (22.2%) | 1.000 | 3 (16.7%) | 5 (27.8%) | 0.691 | 5 (17.9%) | 3 (37.5%) | 0.338 |
|  | Hypomagnesia  (<1.9 mg/dL) | | 0 (0%) | 9 (56.3%) | 9 (19.6%) | 0.001 | 0 (0%) | 9 (25.7%) | 0.089 | 1 (5.9%) | 8 (44.4%) | 0.018 | 4 (15.4%) | 5 (55.6%) | 0.030 |
|  | Hyponatremia  (<135 mmol/L) | | 0 (0%) | 4 (25%) | 4 (8.7%) | 0.011 | 0 (0%) | 4 (11.4%) | 0.559 | 1 (5.9%) | 3 (16.7%) | 0.603 | 2 (7.7%) | 2 (22.2%) | 0.268 |
|  | Hypocalcemia (ionized;  <1.10 mmol/L) | | 3 (10%) | 2 (13.3%) | 5 (11.1%) | 1.000 | 0 (0%) | 5 (14.7%) | 0.313 | 3 (17.7%) | 2 (11.8%) | 1.000 | 4 (15.4%) | 1 (12.5%) | 1.000 |
|  | Hypokalemia  (<3.5 mmol/L) | | 0 (0%) | 7 (43.8%) | 7 (15.2%) | <0.001 | 0 (0%) | 7 (20%) | 0.171 | 1 (5.9%) | 6 (33.3%) | 0.088 | 5 (19.2%) | 2 (22.2%) | 1.000 |
|  | BUN:Creatinine Ratio <10.0 | | 7 (23.3%) | 7 (46.7%) | 14 (31.1%) | 0.111 | 3 (27.3%) | 11 (32.4%) | 1.000 | 5 (31.3%) | 6 (33.3%) | 1.000 | 6 (24%) | 5 (55.6%) | 0.111 |
| **Current or Former User of** | | | |  |  |  |  |  |  |  |  |  |  |  |  |
|  | Cigarettes | | 25 (69.4%) | 3 (17.7%) | 28 (52.8%) | 0.001 | 10 (66.7%) | 18 (47.4%) | 0.237 | 13 (65.0%) | 5 (27.8%) | 0.028 | 15 (51.7%) | 3 (33.3%) | 0.454 |
|  | Alcohol | | 30 (83.3%) | 10 (58.8%) | 40 (75.5%) | 0.086 | 13 (86.7%) | 27 (71.1%) | 0.305 | 16 (80.0%) | 11 (61.1%) | 0.288 | 21 (72.4%) | 6 (66.7%) | 1.000 |
|  | Lija (local alcohol) | | 7 (19.4%) | 0 (0%) | 7 (13.5%) | 0.085 | 1 (6.7%) | 6 (16.2%) | 0.658 | 6 (30.0%) | 0 (0%) | 0.022 | 6 (21.4%) | 0 (0%) | 0.302 |
|  | Illicit Drugs | | 5 (13.9%) | 1 (5.9%) | 6 (11.3%) | 0.651 | 0 (0%) | 6 (15.8%) | 0.167 | 4 (20.0%) | 2 (11.1%) | 0.663 | 5 (17.2%) | 1 (11.1%) | 1.000 |
|  | Traditional/Herbal Medicines | | 11 (30.6%) | 1 (6.7%) | 12 (23.5%) | 0.083 | 5 (33.3%) | 7 (19.4%) | 0.302 | 6 (30.0%) | 1 (6.3%) | 0.104 | 7 (25.0%) | 0 (0%) | 0.309 |
| *As documented on clinical Case Report Form or reported during patient interview | | | | | | |  |  |  |  |  |  |  |  |  |
| ^¥^Parent or Grandparent | | |  |  |  |  |  |  |  |  |  |  |  |  |  |
| *Includes both Objective and Subjective data | | | | |  |  |  |  |  |  |  |  |  |  |  |
