# Supplementary material for: Evidence of nickel and other trace elements and their relationship to clinical findings in acute Mesoamerican Nephropathy: A case-control analysis
Source: PLoS One. 2020 Nov 10;15(11):e0240988. doi: 10.1371/journal.pone.0240988 (PMC7654766; doi:10.1371/journal.pone.0240988)
Supplement: S4 Table — (DOCX) [file pone.0240988.s006.docx]

| **S4 Table. Correlations between trace elements Toenail concentrations (mg/kg dry nail mass) (n=54)** | | | | | | | | | | | | | | |
| --- | --- | --- | --- | --- | --- | --- | --- | --- | --- | --- | --- | --- | --- | --- |
| **KEY to Cell Contents** | **Nickel** | **Aluminum** | **Vanadium** | **Manganese** | **Iron** | **Cobalt** | **Copper** | **Zinc** | **Arsenic** | **Selenium** | **Cadmium** | **Mercury** | **Lead** | **Uranium** |
| SPEARMANS correlation coefficient for Log_10_-transformed concentrations |  |  |  |  |  |  |  |  |  |  |  |  |  |  |
| p-value |  |  |  |  |  |  |  |  |  |  |  |  |  |  |
| Goodman and Kruskal's gamma for correlation of concentrations ≥MDL |  |  |  |  |  |  |  |  |  |  |  |  |  |  |
| Pearson’s Χ^2^/Fisher’s exact p-value for detected presence |  |  |  |  |  |  |  |  |  |  |  |  |  |  |
| Number of Samples ≥MDL |  |  |  |  |  |  |  |  |  |  |  |  |  |  |
| **Aluminum** | 0.4291 | - |  |  |  |  |  |  |  |  |  |  |  |  |
|  | 0.673 |  |  |  |  |  |  |  |  |  |  |  |  |  |
|  | n/a |  |  |  |  |  |  |  |  |  |  |  |  |  |
|  | n/a |  |  |  |  |  |  |  |  |  |  |  |  |  |
|  | 39 |  |  |  |  |  |  |  |  |  |  |  |  |  |
| **Vanadium** | 0.3660 | 0.7982 | - |  |  |  |  |  |  |  |  |  |  |  |
|  | 1.000 | 0.001 |  |  |  |  |  |  |  |  |  |  |  |  |
|  | 0.5833 | n/a |  |  |  |  |  |  |  |  |  |  |  |  |
|  | 0.069 | n/a |  |  |  |  |  |  |  |  |  |  |  |  |
|  | 19 | 22 |  |  |  |  |  |  |  |  |  |  |  |  |
| **Manganese** | 0.3868 | 0.7110 | 0.7170 | - |  |  |  |  |  |  |  |  |  |  |
|  | 1.000 | <0.001 | 0.058 |  |  |  |  |  |  |  |  |  |  |  |
|  | -0.2308 | n/a | -0.4063 |  |  |  |  |  |  |  |  |  |  |  |
|  | 1.000 | n/a | 0.388 |  |  |  |  |  |  |  |  |  |  |  |
|  | 35 | 49 | 19 |  |  |  |  |  |  |  |  |  |  |  |
| **Iron** | 0.3796 | 0.7268 | 0.7128 | 0.5536 | - |  |  |  |  |  |  |  |  |  |
|  | 1.000 | 0.000 | 0.021 | 0.006 |  |  |  |  |  |  |  |  |  |  |
|  | 0.7079 | n/a | 1.0000 | 0.7091 |  |  |  |  |  |  |  |  |  |  |
|  | 0.183 | n/a | 0.262 | 0.257 |  |  |  |  |  |  |  |  |  |  |
|  | 38 | 51 | 22 | 47 |  |  |  |  |  |  |  |  |  |  |
| **Cobalt** | 0.7955 | 0.6903 | n/a | 0.5238 | 0.7609 | - |  |  |  |  |  |  |  |  |
|  | 1.000 | 1.000 | n/a | 1.000 | 1.000 |  |  |  |  |  |  |  |  |  |
|  | 0.6154 | n/a | -0.7830 | -0.0526 | 1.0000 |  |  |  |  |  |  |  |  |  |
|  | 0.252 | n/a | 0.036 | 1.000 | 1.000 |  |  |  |  |  |  |  |  |  |
|  | 9 | 10 | 1 | 9 | 10 |  |  |  |  |  |  |  |  |  |
| **Copper** | 0.2701 | 0.5189 | 0.4970 | 0.4219 | 0.3847 | 0.6802 | - |  |  |  |  |  |  |  |
|  | 1.000 | 0.006 | 1.000 | 0.266 | 0.557 | 1.000 |  |  |  |  |  |  |  |  |
|  | n/a | n/a | n/a | n/a | n/a | n/a |  |  |  |  |  |  |  |  |
|  | n/a | n/a | n/a | n/a | n/a | n/a |  |  |  |  |  |  |  |  |
|  | 39 | 54 | 22 | 49 | 51 | 10 |  |  |  |  |  |  |  |  |
| **Zinc** | -0.1081 | 0.0762 | -0.1716 | 0.1854 | 0.1705 | -0.3176 | 0.6847 | - |  |  |  |  |  |  |
|  | 1.000 | 1.000 | 1.000 | 1.000 | 1.000 | 1.000 | 0.000 |  |  |  |  |  |  |  |
|  | n/a | n/a | n/a | n/a | n/a | n/a | n/a |  |  |  |  |  |  |  |
|  | n/a | n/a | n/a | n/a | n/a | n/a | n/a |  |  |  |  |  |  |  |
|  | 39 | 54 | 22 | 49 | 51 | 10 | 54 |  |  |  |  |  |  |  |
| **Arsenic** | -0.1795 | -0.0364 | 0.3312 | 0.0902 | 0.4583 | 0.3468 | 0.4904 | 0.2519 | - |  |  |  |  |  |
|  | 1.000 | 1.000 | 1.000 | 1.000 | 1.000 | 1.000 | 1.000 | 1.000 |  |  |  |  |  |  |
|  | 0.7213 | n/a | 0.8056 | 0.4679 | 1.0000 | 0.0286 | n/a | n/a |  |  |  |  |  |  |
|  | 0.028 | n/a | <0.001 | 0.638 | 0.274 | 1.000 | n/a | n/a |  |  |  |  |  |  |
|  | 19 | 21 | 15 | 20 | 21 | 4 | 21 | 21 |  |  |  |  |  |  |
| **Selenium** | 0.2287 | -0.0354 | 0.0380 | 0.0851 | 0.4661 | 1.0000 | 0.3793 | 0.0771 | 0.7415 | - |  |  |  |  |
|  | 1.000 | 1.000 | 1.000 | 1.000 | 1.000 | <0.001 | 1.000 | 1.000 | 0.106 |  |  |  |  |  |
|  | 0.7670 | n/a | 0.9937 | -0.3701 | 1.0000 | -0.5701 | n/a | n/a | 0.8448 |  |  |  |  |  |
|  | 0.012 | n/a | <0.001 | 0.640 | 0.253 | 0.161 | n/a | n/a | <0.001 |  |  |  |  |  |
|  | 21 | 23 | 21 | 20 | 23 | 2 | 23 | 23 | 16 |  |  |  |  |  |
| **Cadmium** | 0.5719 | 0.3112 | 0.3261 | 0.3356 | 0.5160 | 0.6471 | 0.3286 | -0.0982 | 0.5039 | 0.4799 | - |  |  |  |
|  | 0.884 | 1.000 | 1.000 | 1.000 | 1.000 | 1.000 | 1.000 | 1.000 | 1.000 | 1.000 |  |  |  |  |
|  | 0.8729 | n/a | 0.5082 | 0.4679 | 0.1266 | 0.2727 | n/a | n/a | 0.8367 | 0.6842 |  |  |  |  |
|  | 0.004 | n/a | 0.050 | 0.638 | 1.000 | 0.486 | n/a | n/a | <0.001 | 0.004 |  |  |  |  |
|  | 20 | 21 | 12 | 20 | 20 | 5 | 21 | 21 | 15 | 14 |  |  |  |  |
| **Mercury** | -0.0415 | -0.0084 | -0.0280 | -0.1243 | -0.1843 | 0.4389 | -0.3574 | -0.4760 | 0.0130 | -0.1912 | 0.0495 | - |  |  |
|  | 1.000 | 1.000 | 1.000 | 1.000 | 1.000 | 1.000 | 1.000 | 0.713 | 1.000 | 1.000 | 1.000 |  |  |  |
|  | 0.6000 | n/a | 0.7360 | 0.3701 | 0.4815 | 0.3208 | n/a | n/a | 0.8045 | 0.8476 | 0.7048 |  |  |  |
|  | 0.035 | n/a | 0.005 | 0.640 | 0.569 | 0.489 | n/a | n/a | 0.002 | <0.001 | 0.010 |  |  |  |
|  | 26 | 31 | 18 | 29 | 30 | 7 | 31 | 31 | 18 | 20 | 17 |  |  |  |
| **Lead** | 0.0090 | 0.2616 | 0.4348 | 0.4128 | 0.3544 | n/a | 0.1416 | -0.0763 | 0.4365 | -0.0003 | 0.3765 | -0.1913 | - |  |
|  | 1.000 | 1.000 | 1.000 | 1.000 | 1.000 | n/a | 1.000 | 1.000 | 1.000 | 1.000 | 0.569 | 1.000 |  |  |
|  | 0.7213 | n/a | 0.9936 | -0.4419 | 1.0000 | -1.0000 | n/a | n/a | 0.7627 | 0.9714 | 0.6710 | 0.7045 |  |  |
|  | 0.028 | n/a | <0.001 | 0.366 | 0.274 | 0.004 | n/a | n/a | 0.001 | <0.001 | 0.010 | 0.010 |  |  |
|  | 19 | 21 | 20 | 18 | 21 | 0 | 21 | 21 | 14 | 19 | 13 | 17 |  |  |
| **Uranium** | 0.3083 | 0.8359 | 0.9547 | 0.8541 | 0.7914 | 1.0000* | 0.4616 | -0.4372 | 0.2742 | -0.2529 | 0.3172 | 0.1790 | 0.2142 | - |
|  | 1.000 | 1.000 | 0.318 | 0.729 | 1.000 | <0.001 | 1.000 | 1.000 | 1.000 | 1.000 | 1.000 | 1.000 | 1.000 |  |
|  | 1.0000 | n/a | 0.6981 | 1.0000 | 1.0000 | 0.2258 | n/a | n/a | 1.0000 | 0.8584 | 1.0000 | 1.0000 | 0.7222 |  |
|  | 0.089 | n/a | 0.051 | 1.000 | 1.000 | 0.632 | n/a | n/a | <0.001 | 0.008 | <0.001 | 0.015 | 0.045 |  |
|  | 8 | 8 | 6 | 8 | 8 | 2 | 8 | 8 | 8 | 7 | 8 | 8 | 6 |  |
| **Chromium** | 0.8207 | 0.6062 | 0.5964 | 0.7597 | 0.6548 | n/a | 0.4991 | 0.1092 | 0.1736 | 0.0698 | 0.7862 | -0.1914 | 0.1559 | 0.8173 |
|  | 0.062 | 1.000 | 1.000 | 0.436 | 1.000 | n/a | 1.000 | 1.000 | 1.000 | 1.000 | 0.431 | 1.000 | 1.000 | 1.000 |
|  | 1.0000 | n/a | 0.8750 | 0.1294 | 1.0000 | -0.5429 | n/a | n/a | 0.8018 | 0.8601 | 0.8892 | 0.8657 | 0.8892 | 0.7757 |
|  | 0.011 | n/a | 0.001 | 1.000 | 1.000 | 0.420 | n/a | n/a | 0.002 | 0.001 | <0.001 | 0.004 | <0.001 | 0.015 |
|  | 13 | 13 | 11 | 12 | 13 | 1 | 13 | 13 | 10 | 11 | 11 | 12 | 11 | 5 |

| ^±^Log10 Concentration |
| --- |
